# Prognostic impact of restored sinus rhythm in patients with sepsis and new-onset atrial fibrillation

**DOI:** 10.1186/s13054-016-1548-2

**Published:** 2016-11-18

**Authors:** Wen Cheng Liu, Wen Yu Lin, Chin Sheng Lin, Han Bin Huang, Tzu Chiao Lin, Shu Meng Cheng, Shih Ping Yang, Jung Chung Lin, Wei Shiang Lin

**Affiliations:** 1 Division of Cardiology, Department of Internal medicine, Tri-Service General Hospital, National Defense Medical Center, No. 325, Section 2, Cheng-Gong Road, Neihu 114, Taipei, Taiwan; 2 School of Public Health, National Defense Medical Center, Taipei, Taiwan; 3 Division of infectious Diseases and Tropical Medicine, Department of Internal medicine, Tri-Service General Hospital, National Defense Medical Center, Taipei, Taiwan

**Keywords:** New-onset atrial fibrillation, Sepsis, Mortality

## Abstract

**Background:**

New-onset atrial fibrillation (NeOAF) is a common type of tachyarrhythmia in critically ill patients and is associated with increased mortality in patients with sepsis. However, the prognostic impact of restored sinus rhythm (SR) in septic patients with NeOAF remains unclear.

**Methods:**

A total of 791 patients with sepsis, who were admitted to a medical intensive care unit from January 2011 to January 2014, were screened. NeOAF was detected by continuous electrocardiographic monitoring. Patients were categorized into three groups: no NeOAF, NeOAF with restored SR (NeOAF to SR), and NeOAF with failure to restore SR (NeOAF to atrial fibrillation (AF)). The endpoint of this study was in-hospital mortality. Patients with pre-existing AF were excluded.

**Results:**

We reviewed the data of 503 eligible patients, including 263 patients with no NeOAF and 240 patients with NeOAF. Of these 240 patients, SR was restored in 165 patients, and SR could not be restored in 75 patients. The NeOAF to AF group had the highest in-hospital mortality rate of 61.3% compared with the NeOAF to SR and no NeOAF groups (26.1% and 17.5%, respectively). Moreover, multivariate logistic regression analysis revealed that failure of restored SR was independently associated with increased in-hospital mortality in patients with sepsis and NeOAF.

**Conclusions:**

Failure to restore a sinus rhythm in patients with new-onset atrial fibrillation may be associated with increased in-hospital mortality in patients with sepsis. Further prospective studies are needed to clarify the effects of restoration of sinus rhythm on survival in patients with sepsis and new-onset atrial fibrillation.

**Electronic supplementary material:**

The online version of this article (doi:10.1186/s13054-016-1548-2) contains supplementary material, which is available to authorized users.

## Background

Atrial fibrillation (AF) is the most common sustained cardiac arrhythmia in critically ill patients [1, 2]. In past decades, increased attention has been paid to new-onset atrial fibrillation (NeOAF) in patients undergoing cardiac or noncardiac surgery, patients with major trauma, and critically ill patients, and it is associated with poor prognosis [3–10].

Sepsis, a potentially life-threatening organ dysfunction syndrome caused by a dysregulated host response to infection, is one of the leading causes of death worldwide. On the basis of disease severity, it can be categorized into sepsis and septic shock. Septic shock is defined by requirement for vasopressors to maintain a mean arterial pressure of 65 mmHg or greater and serum lactate level greater than 2 mmol/L in the absence of hypovolemia [11–13]. In recent studies, NeOAF has been shown to be prevalent in patients with sepsis and to be associated with longer hospital stay and increased morbidity and mortality [14–24].

A longer AF burden leads to worsening cardiac effects and makes sepsis treatment more difficult. A previous study found that management with electrical cardioversion or pharmacological treatment for NeOAF may improve outcomes in critically ill patients [18]. It remains unclear whether restored sinus rhythm (SR) in patients with sepsis and NeOAF is associated with favorable prognosis. Therefore, among patients with sepsis, this study investigated the impact on in-hospital mortality of restoration of SR in patients with NeOAF in comparison with failure to restore SR in those with NeOAF and without NeOAF.

## Methods

### Study population

This single-center, retrospective, comparative cohort study was conducted at Tri-Service General Hospital, National Defense Medical Center, and involved screening of consecutive adults who were admitted to a medical intensive care unit (ICU) with a diagnosis of sepsis or septic shock from January 2011 to January 2014. The institutional review board of the center approved the study with a protocol number of 2-104-05-003. Informed consent was waived due to the observational design.

**Fig. 1:**
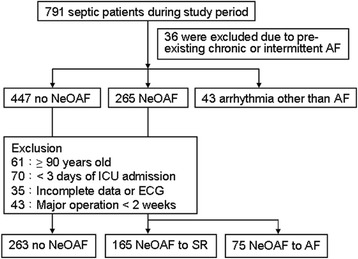
Patients with sepsis were enrolled and categorized into groups based on the occurrence of atrial fibrillation (*AF*) and AF status during ICU stay from January 2011 to January 2014. *ECG* electrocardiogram, *SR* sinus rhythm, *NeOAF* new-onset atrial fibrillation

Previous medical records and a 12-lead electrocardiogram (ECG) were fully evaluated by chart review. Pre-existing AF and arrhythmia other than AF were excluded initially. Then, individuals meeting any of the following criteria were excluded: (1) age older than 90 years; (2) ICU stay less than 3 days; (3) missing or incomplete clinical data or ECG; or (4) major surgery within 2 weeks prior to this admission. A flowchart of the enrollment of the study population is shown in Fig. 1.

Medical records were reviewed to collect information on baseline characteristics including demographic data, laboratory parameters, and the underlying comorbidities of hypertension, diabetes mellitus (DM), coronary artery disease (CAD), heart failure, cerebrovascular disease (CVD), chronic obstructive pulmonary disease (COPD), chronic kidney disease (CKD), and prior thyroid disease. Prior medication use, including beta-blockers or non-dihydropyridine calcium channel blockers (non-DHP CCBs) was evaluated. Transthoracic echocardiographic findings were assessed on ICU admission with a focus on left atrium diameter (LAD), left ventricular end-diastolic dimension (LVEDD), left ventricular end-systolic dimension (LVESD), and left ventricular ejection fraction (LVEF).

### Diagnosis of sepsis and its severity

Sepsis and septic shock were defined based on the The Third International Consensus Definitions for Sepsis and Septic shock (Sepsis-3) [13]. The management of sepsis was based on early goal-directed therapy recommended in the guidelines of the Surviving Sepsis Campaign [11, 12].

According to Surviving Sepsis Campaign recommendations, information on infection sites and sepsis-induced acute organ dysfunction, including neurologic, circulatory, respiratory, renal, hepatic, and hematologic dysfunction was collected. Scores of severity-of-disease classification systems including the Acute Physiology and Chronic Health Evaluation II (APACHE II) and Sequential Organ Failure Assessment (SOFA) were recorded for each individual on ICU admission [25, 26].

### Detection and management of NeOAF

During ICU admission, all patients received continuous electrocardiographic monitoring. The 12-lead ECGs were obtained when deemed clinically appropriate by the critical care team. The diagnosis of AF, defined as the absence of P waves and irregular ventricular activity lasting for more than 30 seconds, was confirmed by clinicians. After NeOAF was detected, the decision of electrical cardioversion, pharmacological treatment, or a wait-and-see strategy for NeOAF was made by the responsible clinicians based on the patient’s hemodynamic status and contraindication for antiarrhythmic agents.

Failure to restore SR in patients with NeOAF was defined as persistent or recurrent AF 7 days after the onset of NeOAF and was referred to as NeOAF to AF. The restoration of SR in patients with NeOAF is referred to as NeOAF to SR. Patients with NeOAF who died within 7 days after admission was categorized as the indicated group based on the final rhythm before death. The endpoint of this study was in-hospital mortality, which was confirmed by records of the death note.

### Statistical analyses

Statistical analyses were performed using the SPSS software package (version 17.0; SPSS, Chicago, IL, USA), and differences were considered statistically significant when the *P* value was <0.05. Continuous variables are presented as mean and standard deviation. Categorical variables are presented as the number of patients and the corresponding percentage. The differences in the characteristics of the groups were assessed using the unpaired two-tailed Student *t* test or one-way analysis of variance (ANOVA) for continuous variables and the chi-square and Fisher exact tests for nominal variables.

The significant variables (*P* < 0.05) associated with the individual AF status in patients were identified. Backward stepwise logistic regression analysis was conducted to evaluate the significant predictors of NeOAF and NeOAF to AF. To evaluate the association between the NeOAF status and in-hospital mortality, we constructed two regression models. Model 1 was the univariate logistic regression analysis of the AF status. Model 2 was the multivariate logistic regression analysis after adjustment for significant confounding factors, which were selected based on the criteria of being associated with exposure, associated with outcomes, and not intermediate variables between exposure and outcome [27]. A Cox proportional hazard model with time-varying exposure was constructed to confirm the associations between individual AF status and in-hospital mortality.

## Results

### Study population and incidence of NeOAF

A total of 791 patients were screened. Of these patients, 36 and 43 patients were excluded due to pre-existing AF and arrhythmia other than AF, respectively. The prevalence of pre-existing AF was 4.5% (36/791). The incidence of NeOAF was 35.1% (265/755). After excluding patients older than 90 years, with less than 3 days of ICU stay, missing or incomplete data, or having had major surgery within 2 weeks of sepsis, 503 eligible patients were evaluated based on the different AF status. Among these, 263 patients had no AF and 240 patients developed NeOAF after ICU admission. SR was restored in 165 patients (NeOAF to SR) and SR could not be restored in 75 patients (NeOAF to AF) as shown in Fig. 1.

### Baseline characteristics of patients with sepsis and different AF status

The baseline characteristics of patients with sepsis and different AF status are compared in Table 1. Patients in the NeOAF to SR and NeOAF to AF groups were significantly older than those in the no NeOAF group. Moreover, a higher prevalence of hypertension, heart failure, CAD, and CVD was observed in patients with NeOAF compared with those without NeOAF. No significant differences were observed in COPD, DM, uremia, and thyroid disorder between these three groups. Regarding prior medication use before ICU admission, patients with the NeOAF to AF group had lower beta-blocker use compared with those with the NeOAF to SR group.

**Table 1:** Baseline characteristics of patients with sepsis and different AF status

Characteristics	Overall patients (*n* = 503)			*P* value
	No NeOAF (*n* = 263)	NeOAF to SR (*n* = 165)	NeOAF to AF (*n* = 75)	
Age, years	69.5 ± 15.6	77.8 ± 10.3†	76.2 ± 11.0^a^	<0.01
Male, *n* (%)	174 (66.2)	90 (54.5)†	46 (61.3)	0.06
Comorbidities, *n* (%)				
Hypertension	145 (55.1)	111 (67.3)†	44 (58.7)	0.04
Heart failure	25 (9.5)	35 (21.2)†	15 (20.0)^a^	<0.01
Coronary artery disease	90 (34.2)	70 (42.4)	37 (49.3)^a^	0.04
Cerebrovascular disease	56 (21.3)	53 (32.1)†	23 (30.7)	0.03
COPD	52 (19.8)	28 (17.0)	12 (16.0)	0.66
Diabetes mellitus	97 (36.9)	65 (39.4)	21 (28.0)	0.23
Uremia	18 (6.8)	17 (10.3)	9 (12.0)	0.26
Thyroid disorder	10 (3.8)	10 (6.1)	6 (8.0)	0.29
Prior medication, *n* (%)				
Beta blocker	29 (11.0)	27 (16.4)	4 (5.3)^b^	0.04
Calcium channel blocker	9 (3.4)	10 (6.1)	2 (2.7)	0.32

*COPD* chronic obstructive pulmonary disease, *AF* atrial fibrillation, *NeOAF* new-onset atrial fibrillation. *SR* sinus rhythm. ^a^ *P* < 0.05 vs. no NeOAF. ^b^ *P* < 0.05 vs. NeOAF to SR

Table 2 shows the laboratory and echocardiographic results in patients with sepsis and different AF status. All the laboratory data and examinations were performed at the beginning of ICU admission. No statistically significant differences were observed in serum white blood cell (WBC) count, electrolytes, albumin, or B-type natriuretic peptide (BNP) levels among the three groups. However, C-reactive protein (CRP) was lowest in the NeOAF to SR group than in the no NeOAF and NeOAF to AF groups (10.0 ± 9.9, 12.8 ± 10.9 and 11.2 ± 8.5 mmol/L, respectively). Troponin-I was highest in the NeOAF to AF group than in the no NeOAF and NeOAF to SR groups. On echocardiography, both the NeOAF to SR and NeOAF to AF group had a larger left atrial diameter than the no NeOAF group. Left ventricular systolic dysfunction was more prevalent in patients with NeOAF to AF.

**Table 2:** Laboratory findings and echocardiography index at admission in patients with sepsis and different AF status

	Overall patients (*n* = 503)			*P* value
	No NeOAF (*n* = 263)	NeOAF to SR (*n* = 165)	NeOAF to AF (*n* = 75)	
Laboratory				
WBC (×10^3^/L)	13.47 ± 8.73	13.43 ± 7.23	15.39 ± 11.73	0.22
CRP (mmol/L)	12.8 ± 10.9	10.0 ± 9.9†	11.2 ± 8.5	0.02
Na^+^ (mmol/L)	135.6 ± 8.5	135.7 ± 10.1	136.7 ± 7.8	0.64
K^+^ (mmol/L)	4.1 ± 1.0	4.2 ± 1.0	4.1 ± 0.9	0.46
Free Ca^2+^ (mmol/L)	4.23 ± 0.31	4.35 ± 0.46	4.17 ± 0.68	0.46
Albumin (g/dL)	2.7 ± 0.6	2.7 ± 0.6	2.6 ± 0.6	0.37
BNP (pg/ml)	918.6 ± 1152.2	1131.3 ± 1325.6	1102.4 ± 1418.1	0.58
Tr-I (ng/ml)	1.09 ± 5.92	1.98 ± 5.95	3.71 ± 13.42^a^	0.04
Echocardiography				
LAD (mm)	36.5 ± 7.4	38.4 ± 7.2†	40.5 ± 7.2^a^	<0.01
LVEDD (mm^3^)	45.7 ± 8.0	46.1 ± 7.7	46.6 ± 7.5	0.73
LVESD (mm^3^)	30.3 ± 8.0	30.9 ± 7.3	32.3 ± 8.4	0.22
LVEF < 50%, *n* (%)	31 (13.7)	29 (18.2)	18 (27.7)†	0.03

*NeOAF* new-onset atrial fibrillation, *AF* atrial fibrillation, *SR* sinus rhythm, *WBC* white blood cell count, *CRP* C-reactive protein, *BNP* B-type natriuretic peptide, *Tr-I* troponin I, *LAD* left atrium diameter, *LVEDD* left ventricular end diastolic diameter, *LVESD* left ventricular end systolic diameter, *LVEF* left ventricular ejection fraction. ^a^ *P* < 0.05 vs. no NeOAF

### Sepsis severity in patients with different AF status

The most common infection site of sepsis in our study population was the respiratory tract, followed by the urinary tract and intra-abdominal sites (Table 3). Patients with NeOAF had significantly high SOFA and APACHE II scores, which are severity indices of sepsis. The no NeOAF, NeOAF to SR, and NeOAF to AF groups had SOFA scores of 7.0 ± 3.2, 7.6 ± 3.0, and 9.3 ± 3.2 and APACHE II scores of 21.6 ± 5.5, 22.8 ± 5.8, and 24.6 ± 6.1, respectively. Dopamine and norepinephrine were more commonly used in the NeOAF to AF group.

**Table 3:** Disease severity index of sepsis in patients with various AF statuses

	Overall patients (*n* = 503)			*P* value
	No NeOAF (*n* = 263)	NeOAF to SR (*n* = 165)	NeOAF to AF (*n* = 75)	
Infection site, *n* (%)				
Respiratory tract	168 (63.9)	112 (67.9)	48 (64.0)	
Urinary tract	57 (21.7)	35 (21.2)	14 (18.7)	
Gastrointestinal	23 (8.7)	9 (5.5)	5 (6.7)	
Others	15 (5.7)	9 (5.5)	8 (10.7)	
SOFA score	7.0 ± 3.2	7.6 ± 3.0	9.3 ± 3.2^bc^	<0.01
APACHE II score	21.6 ± 5.5	22.8 ± 5.8	24.6 ± 6.1^b^	<0.01
Total organ failure^a^, *n*	2 (1–3)	2 (1–3)	3 (2–4)	
Neurologic failure	87 (33.1)	49 (29.7)	39 (52.0)^bc^	<0.01
Circulatory failure	118 (44.9)	82 (49.7)	57 (76.0)^bc^	<0.01
Respiratory failure	229 (87.1)	150 (90.9)	71 (94.7)	0.13
Hepatic failure	8 (3.0)	4 (2.4)	8 (10.7)^bc^	<0.01
Renal failure	86 (32.7)	65 (39.4)	39 (52.0)^b^	<0.01
Hematologic failure	9 (3.4)	5 (3.0)	4 (5.3)	0.66
Vasopressor use, *n* (%)				
Dopamine use	95 (36.3)	64 (38.8)	49 (65.3)^bc^	<0.01
Norepinephrine use	80 (30.4)	58 (35.2)	48 (64.0)^bc^	<0.01
Intervention, *n* (%)				
Ventilator use	221 (84.0)	143 (86.7)	69 (92.0)	0.21
New-onset dialysis	50 (19.0)	35 (21.2)	19 (25.3)	0.48

*NeOAF* new-onset atrial fibrillation, *AF* atrial fibrillation, *SR* sinus rhythm *SOFA* Sequential Organ Failure Assessment score, *APACHE II* Acute Physiology and Chronic Health Evaluation II. ^a^Total organ failure is presented as median and interquartile range. ^b^ *P* < 0.05 vs. no NeOAF. ^c^ *P* < 0.05 vs. NeOAF to SR

### Management of NeOAF in patients with sepsis

We reviewed 240 eligible patients with NeOAF. Among these 240 patients, SR was restored in 165 patients (NeOAF to SR), and SR could not be restored in 75 patients (NeOAF to AF). Treatments received by patients with NeOAF are shown in Table 4. Beta-blockers (36.7%) and amiodarone (33.3%) were the most commonly used in patients with NeOAF and sepsis, followed by non-DHP CCBs and digitalis glycosides. Electrical cardioversion was performed in only eight patients. There were no significant differences in pharmacological therapies and electrical cardioversion between the NeOAF to SR and NeOAF to AF groups.

**Table 4:** Therapeutic interventions in patients with sepsis and NeOAF

	NeOAF patients (*n* = 240)			*P* value
	Overall (*n* = 240)	NeOAF to SR (*n* = 165)	NeOAF to AF (*n* = 75)	
Pharmacological, *n* (%)				
Amiodarone	80 (33.3)	52 (31.5)	28 (37.3)	0.38
Beta-blockers	88 (36.7)	67 (40.6)	21 (28.0)	0.06
Non-DHP CCBs^a^	66 (27.5)	47 (28.5)	19 (25.3)	0.61
Digoxin glycosides	27 (11.3)	15 (9.1)	12 (16.0)	0.12
Electrical cardioversion, *n* (%)	8 (3.3)	4 (2.4)	4 (5.3)	0.25

*NeOAF* new-onset atrial fibrillation, *AF* atrial fibrillation, *SR* sinus rhythm. ^a^Only indicates non-dihydropyridine calcium channel blockers (non-DHP CCBs)

### Clinical impact of rhythm control in patients with sepsis and NeOAF

**Fig. 2:**
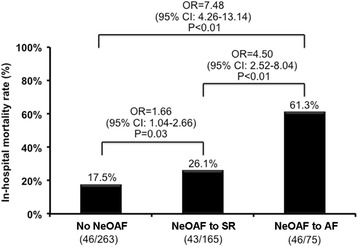
In-hospital mortality in patients with sepsis and different AF status. *NeOAF* new-onset atrial fibrillation, *AF* atrial fibrillation, *SR* sinus rhythm

Patients with sepsis and NeOAF had a longer ICU stay (NeOAF to SR group: 16.7 ± 13.6; NeOAF to AF group: 17.3 ± 23.3 days) than those without NeOAF (11.4 ± 11.1 days). Moreover, the NeOAF to AF group had the highest in-hospital mortality rate of 61.3% (46/75) compared with the rate of 26.1% (43/165) in the NeOAF to SR group and of 17.5% (46/263) in the no NeOAF group (*P* < 0.01) as shown in Fig. 2.

Based on the data in Tables 1, 2, 3, and 4, the significant variables were selected associated with different AF status as follows: age; hypertension; heart failure; CAD; CVD; prior beta-blocker use; CRP; Troponin-I; LA dimension; LVEF; SOFA scores; APACHE II scores; neurological, circulation, or hepatic failure; renal dysfunction; dopamine use; and norepinephrine use. The logistic regression models were constructed to analyze the singificant predictors of NeOAF and NeOAF to AF in patients with sepsis. For all patients with NeOAF, these predictors included age, heart failure, CVD, and SOFA scores. For patients with NeOAF to AF, these predictors included age, LA dimension, APACHE II scores, hepatic dysfunction, dopamine use, and norepinephrine use.

Regarding the association between different AF status and in-hospital mortality, the univariate logistic regression analysis showed that NeOAF to AF was significantly associated with increased in-hospital mortality compared to NeOAF to SR and no NeOAF (OR = 4.50, 95% CI = 2.52–8.04; *P* < 0.01 and OR = 7.48, 95% CI = 4.26–13.14; *P* < 0.01) (Table 5). The multivariate logistic regression analysis, which was adjusted for the significant confounding factors including: age; CAD; LVEF; SOFA scores; neurological, circulation, hepatic, or renal dysfunction; and dopamine and norepinephrine use, revealed that NeOAF to AF itself was an independent risk factor for in-hospital mortality in patients with sepsis compared to NeOAF to SR and no NeOAF (OR = 2.22, 95% CI = 1.02–4.83; *P* < 0.05 and OR = 3.31, 95% CI = 1.54–7.13; *P* < 0.01). Moreover, Cox proportional hazard models were applied to examine the association between different AF status and in-hospital mortality. There were some differences compared with the logistic regression model, especially in the association between NeOAF to AF or no NeOAF and in-hospital mortality (Additional file 1: Table S1). However, the associations between NeOAF to AF or NeOAF to SR and in-hospital mortality were similar (*P* < 0.05).

**Table 5:** Association between different AF status and in-hospital mortality

Model	OR	95% CI of OR	*P* value
Model 1^a^			
NeOAF to SR vs. no NeOAF	1.66	1.04–2.66	0.03
NeOAF to AF vs. no NeOAF	7.48	4.26–13.14	<0.01
NeOAF to AF vs. NeOAF to SR	4.50	2.52–8.04	<0.01
Model 2^b^			
NeOAF to SR vs. no NeOAF	1.49	0.79–2.83	0.22
NeOAF to AF vs. no NeOAF	3.31	1.54–7.13	<0.01
NeOAF to AF vs. NeOAF to SR	2.22	1.02–4.83	0.045

*NeOAF* new-onset atrial fibrillation, *AF* atrial fibrillation, *SR* sinus rhythm, *OR* odds ratio, *CI* confidence interval. ^a^Unadjusted model. ^b^Adjusted for age, coronary artery disease, left ventricular ejection fraction, Sequential Organ Failure Assessment score, neurologic failure, circulatory failure, hepatic failure, renal failure, dopamine use, and norepinephrine use

## Discussion

### Main findings

In this study, we demonstrated that NeOAF in patients with sepsis was associated with an increased risk of in-hospital mortality. Baseline organ dysfunction, especially neurologic, circulatory, hepatic, or renal dysfunction (which are significant confounders for in-hospital mortality and NeOAF status in patients with sepsis), was statistically different in the three groups. Intriguingly, patients with NeOAF in whom SR could not be restored (as NeOAF to AF) has 2.22-fold higher risks of in-hospital mortality than those in whom SR was restored (as NeOAF to SR) and 3.31-fold higher risks of in-hospital mortality than those without NeOAF (as no NeOAF). In addition, in-hospital mortality in patients with NeOAF and restored SR was not statistically different compared with patients with no NeOAF (*P* = 0.22, 95% CI = 0.79–2.83).

Previous research suggests that the prevalence of pre-existing AF among hospitalized patients ranges from 2 to 15%. In our study, the prevalence of pre-existing AF was 4.5%. Pre-existing AF was evaluated by previous medical records and 12-lead ECG at admission, which may underestimate the prevalence of AF in patients with unrecognized paroxysmal AF. Moreover, the incidence of NeOAF was 35.1% in this study, which is higher than previous studies ranging from 10 to 46% [22]. It is probably due to higher disease severity, organ dysfunction and frequent vasopressor use among the enrolled patients.

### Association between sepsis and NeOAF

According to the mechanism of NeOAF proposed by most studies, NeOAF in patients with sepsis is attributable to sepsis-related inflammatory states. Basically, systemic inflammation can alter atrial electrophysiology and structural substrates, leading to increased vulnerability to AF. Complex inflammatory pathways affect myolysis, cardiomyocyte apoptosis, and the activation of fibrotic pathways through fibroblasts, transforming growth factor-β, and matrix metalloproteases, contributing to structural remodeling of the atria [28, 29]. In addition, sepsis-related micro-abscess formation and autonomic dysfunction may be also responsible for the potential mechanisms for the occurrence of NeOAF [30, 31].

The clinical management of patients with sepsis is also associated with increased risks of NeOAF. Previous studies suggest that fluid resuscitation and vasopressor use in response to hypotension greatly contribute to the development of NeOAF [24, 32]. Increased net positive fluid resuscitation may result in an acute increase in left ventricular end-diastolic pressure and subsequent left atrial stretch, providing an anatomical substrate for the occurrence of AF. Vasopressor agents, particularly those with β-adrenergic effects, may also play a direct role in triggering NeOAF [14, 18, 24, 33].

Serum CRP, an acute-phase reactant, has been considered a clinical indicator of inflammation. Hu et al. demonstrated a positive relationship between elevated CRP and the development and maintenance of AF [29]. AF is more common in patients with high CRP than in those with low CRP. Aviles et al. also reported that baseline CRP can be used to predict the risk of AF in the general population [34]. In patients with sepsis, Meierhenrich et al. demonstrated continuous increments in CRP before the onset of NeOAF [18]. These findings support the hypothesis that the systemic inflammatory state can promote and perpetuate AF. However, in our study, slightly higher CRP was observed in patients without NeOAF than in those with NeOAF. It was possibly due to CRP only being measured once at admission, the lack of series follow-up during sepsis, or the development of NeOAF. Therefore, CRP could be influenced by numerous confounding factors during sepsis and we could not demonstrate the positive association between CRP level and NeOAF in our study.

### Hypothesis of rhythm control strategy in patients with sepsis and NeOAF

The development of NeOAF could lead to deteriorated hemodynamic status during sepsis. The occurrence of NeOAF makes the disease more complicated and makes treatment during sepsis or septic shock more challenging because of adverse cardiovascular effects including rapid heart rate, irregular rhythm, loss of atrial systole, and neurohormonal activation. In the acute phase, both tachycardia and loss of atrial systole caused by AF could reduce the cardiac output, further destablizing patients with sepsis. Subsequently, the burden of NeOAF in patients with sepsis may result in acute heart failure [35, 36]. Moreover, atrial stasis and sepsis-related coagulopathy could lead to systemic embolization and an increased risk of ischemia stroke [19].

AF itself has been found to subsequently generate an inflammatory response that further enhances atrial remodeling and perpetuates arrhythmia. Previous studies have proposed that restoration and maintenance of SR in patients with AF could result in gradually decreasing CRP, indicating that AF initiates inflammation [37, 38]. Therefore, inflammation could promote AF and vice versa. To stop the vicious cycle between inflammation and AF, the early management of NeOAF by rhythm control may be beneficial in patients with sepsis.

Our study highlighted that the restoration of SR in patients with sepsis and NeOAF led to more favorable prognosis in comparison with failure to restore SR in these patients. It is evident that restored SR in patients with NeOAF could produce enhanced diastolic filling and a rapid increase in left ventricular systolic performance among patients with NeOAF [39]. Meierhenrich et al. reported that 23 out of 50 surgical patients with septic shock developed NeOAF [18]. They also demonstrated that failure to restore SR was associated with increased ICU mortality (71.4% versus 21.4%). Compared to our study, we conducted a study in a more modest-sized cohort of patients with non-surgical and mixed sepsis and septic shock, and the disease severity of sepsis was relatively lower in our study (SOFA score 7–9 versus 9–12). Consistently, we found that the in-hospital mortality rate was significantly higher in the NeOAF to AF group than in the NeOAF to SR group (61.3% versus 26.1%). Restoration of SR appeared to be a favorable prognostic marker of in-hospital mortality in patients with sepsis and NeOAF, and this might have further clinical implications for NeOAF surveillance and treatment in this settings.

### Treatment recommendation for NeOAF in patients with sepsis

So far, recommendations for management of NeOAF in patients with sepsis are dependent on observational studies or expert opinions [40]. In principle, clinicians should manage potentially reversible AF triggers including electrolyte imbalances, rate-affected medication, myocardial injury, and airway obstruction before starting antiarrhythmic treatment.

Pharmacological options between a rate control and a rhythm control strategy remain uncertain in patients with sepsis and NeOAF. The rate control strategy tolerates AF, but improves ventricular filling and avoids a tachycardia-induced cardiomyopathy. It could be achieved by using atrio-ventricular nodal blocking agents, such as beta-blockers, non-DHP CCBs and digoxin. However, it should be cautiously applied in patients with septic shock because these agents have negative inotropic and vasodilatory effects, probably leading to reduced cardiac output and hypotension. A short-acting beta-blocker with esmolol may be the first consideration in patients with septic shock and NeOAF [41]. In the past, a rhythm control compared with a rate control strategy has shown no survival benefit in non-critically ill patients [42]. Amiodarone can slow nodal conduction and convert AF to sinus rhythm. It has less negative inotropic effects compared with beta-blockers and non-DHP CCB and may be safer in patients with structural heart disease. Nevertheless, current evidence is not strong enough to recommend that amiodarone may improve the prognosis in patients with sepsis and NeOAF [43, 44]. Moreover, as shown in Table 1, the prevalence of previous treatment with beta-blockers or CCBs was higher in the no NeOAF and NeOAF to SR groups compared to the NEOAF to AF group. However, evidence on NeOAF prophylaxis in patients with sepsis is lacking. Further prospective, randomized studies are necessary to clarify the effects of beta-blockers or CCBs on NeOAF prophylaxis in patients with sepsis.

Regarding anticoagulant use in patients with sepsis and NeOAF, some studies have demonstrated that NeOAF is associated with an increased risk of short-term and long-term ischemic stroke, with threefold higher stroke rates in patients with NeOAF than in those without NeOAF during sepsis [19, 45]. However, the benefit and risk of anticoagulant use during an acute stage of sepsis remain unclear. Thus, anticoagulants cannot be currently recommended as a cornerstone treatment for sepsis-related NeOAF, and further large prospective studies are warranted.

### Study limitations

The present study had several limitations. First, it was a single-center, retrospective, cohort study that relied on accurate documentation, restricting the external validity of our result. Second, several baseline characteristics of the three groups were not equal. This might lead to bias related to outcomes to a certain extent, despite statistically adjusting for these confounders. Third, we could not determine which factors were the main factors for the restoration of SR in patients with sepsis and NeOAF. This could be attributed to the effect of antiarrhythmic agents, improvement in sepsis-related inflammation, or spontaneous recovery. Fourth, a fixed protocol was not used for the management of NeOAF. The pharmacological intervention was retrospectively recorded and based on the clinicians’ orders under different clinical conditions. We could not determine which medication led to the therapeutic effect of SR restoration. Fifth, the data on the use of anticoagulation were limited. Sixth, data on the duration of vasopressor use, which may be a confounder in the AF and mortality assessment, were not available in the current study. Seventh, the association between NeOAF to AF or no NeOAF and in-hospital mortality was different in the two analysis models. The discrepancy was due to the different definition of outcome in the logistic regression models (event) and Cox proportional hazard models (time to event). Finally, data on long-term follow up in the NeOAF population, such as length of antiarrhythmic therapy, new strokes, and anticoagulant therapy are not obtainable.

## Conclusion

In summary, NeOAF is prevalent in patients with sepsis and is related to increased in-hospital mortality. We proposed that successful restoration of SR in patients with sepsis and NeOAF may offer a more favorable outcome than in those in whom SR could not be restored. However, a larger, prospective comparative study is needed to elucidate the clinical implications between a rate control and a rhythm control strategy in patients with sepsis and NeOAF.

## Key messages

- New-onset atrial fibrillation (NeOAF) is prevalent and associated with increased mortality in patients with sepsis.
- It remains unclear whether restored sinus rhythm (SR) of NeOAF leads to better outcomes and how to suitably manage in patients with NeOAF.
- NeOAF with failure to restore SR (but not all NeOAF) was an independent risk for in-hospital mortality in patients with sepsis.
- Further prospective trials are warranted to elucidate the clinical implications of a rate versus a rhythm control strategy in patients with sepsis and NeOAF.

## Additional file

Additional file 1: Table S1. Hazard ratio of different AF status for in-hospital mortality. Cox proportional hazard model with time-varying exposure was conducted for the analysis. (DOC 32 kb)

## Abbreviations

AF: atrial fibrillation
APACHE II: Acute Physiology and Chronic Health Evaluation II
CAD: coronary artery disease
CKD: chronic kidney disease
COPD: chronic obstructive pulmonary disease
CPR: C-reactive protein
CVD: cerebrovascular disease
DM: diabetes mellitus
ECG: electrocardiogram
ICU: intensive care unit
LAD: left atrium diameter
LVEDD: left ventricular end-diastolic dimension
LVEF: left ventricular ejection fraction
LVESD: left ventricular end-systolic dimension
NeOAF: new-onset atrial fibrillation
non-DHP CCB: non-dihydropyridine calcium channel blockers
SOFA: Sequential Organ Failure Assessment
SR: sinus rhythm
