# Supplementary material for: Prognostic impact of restored sinus rhythm in patients with sepsis and new-onset atrial fibrillation
Source: Crit Care. 2016 Nov 18;20:373. doi: 10.1186/s13054-016-1548-2 (PMC5114755; doi:10.1186/s13054-016-1548-2)
Supplement: Additional file 1: Table S1. — Hazard ratio of different AF status for in-hospital mortality. Cox proportional hazard model with time-varying exposure was conducted for the analysis. (DOC 32 kb) [file 13054_2016_1548_MOESM1_ESM.doc]

**Table S1 Hazard ratio of various AF statuses with in-hospital mortality**

| Model | HR | 95% CI of HR | P value |
| --- | --- | --- | --- |
| Model 1† |  |  |  |
| NeOAF to SR *vs*. no NeOAF | 1.15 | 0.76- 1.74 | 0.52 |
| NeOAF to AF *vs*. no NeOAF | 3.09 | 2.05-4.67 | <0.01 |
| NeOAF to AF *vs*. NeOAF to SR | 2.70 | 1.77-4.10 | <0.01 |
| Model 2‡ |  |  |  |
| NeOAF to SR *vs*. no NeOAF | 0.80 | 0.52-1.25 | 0.33 |
| NeOAF to AF *vs*. no NeOAF | 1.32 | 0.83-2.10 | 0.24 |
| NeOAF to AF *vs*. NeOAF to SR | 1.64 | 1.04-2.60 | 0.03 |

HR, hazard ratio; CI, confidence interval.

† Unadjusted model.

‡ Adjusted for age, CAD, LVEF, SOFA score, neurologic, circulatory, hepatic, renal dysfunction, dopamine use, and norepinephrine use.
